# Supplementary figures and images for: Suppression of Drug Resistance Reveals a Genetic Mechanism of Metabolic Plasticity in Malaria Parasites
Source: mBio. 2018 Nov 13;9(6):e01193-18. doi: 10.1128/mBio.01193-18 (PMC6234871; doi:10.1128/mBio.01193-18)

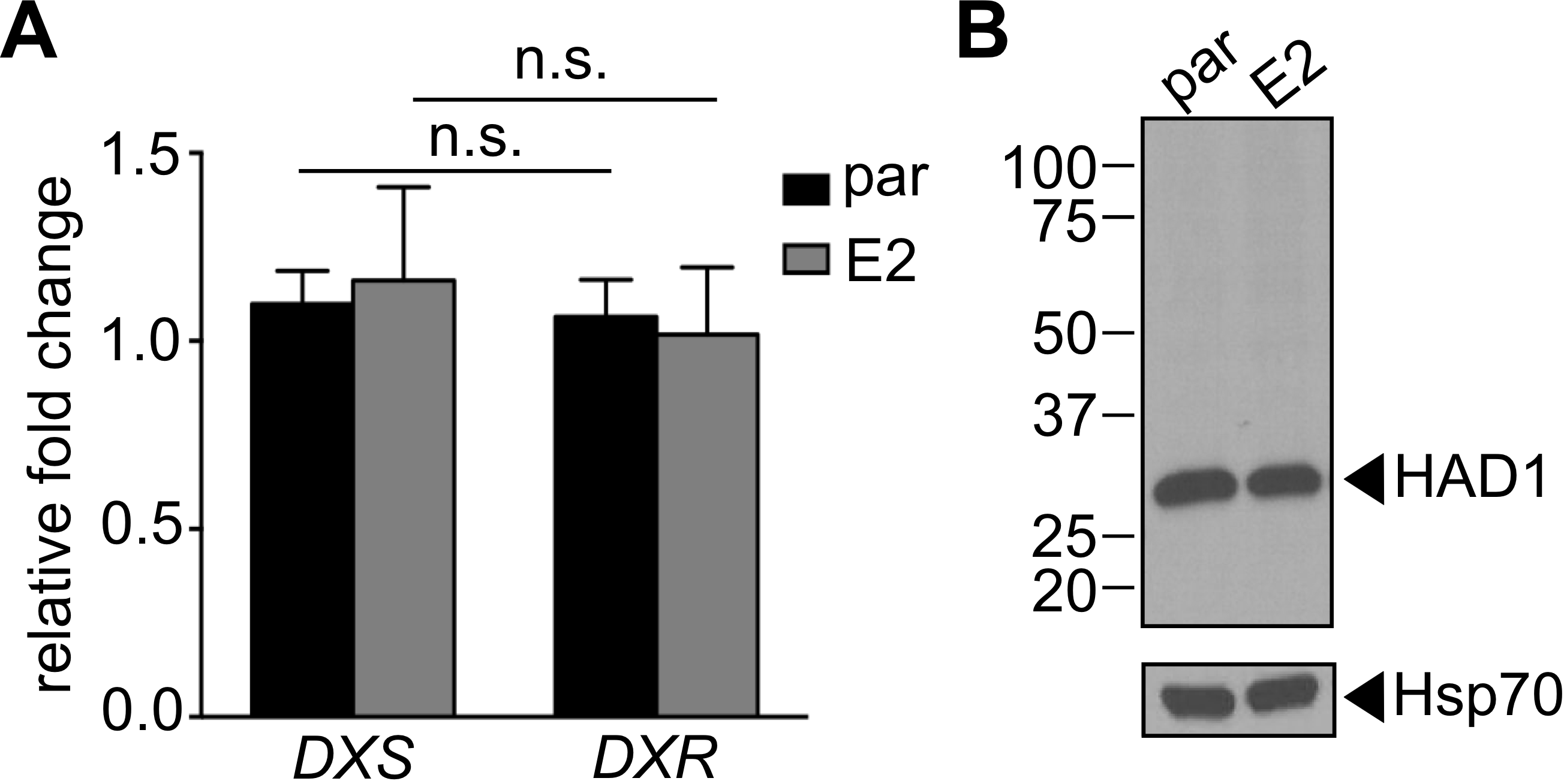

Supplement: FIG S1 [file mbo006184175sf1.tif]

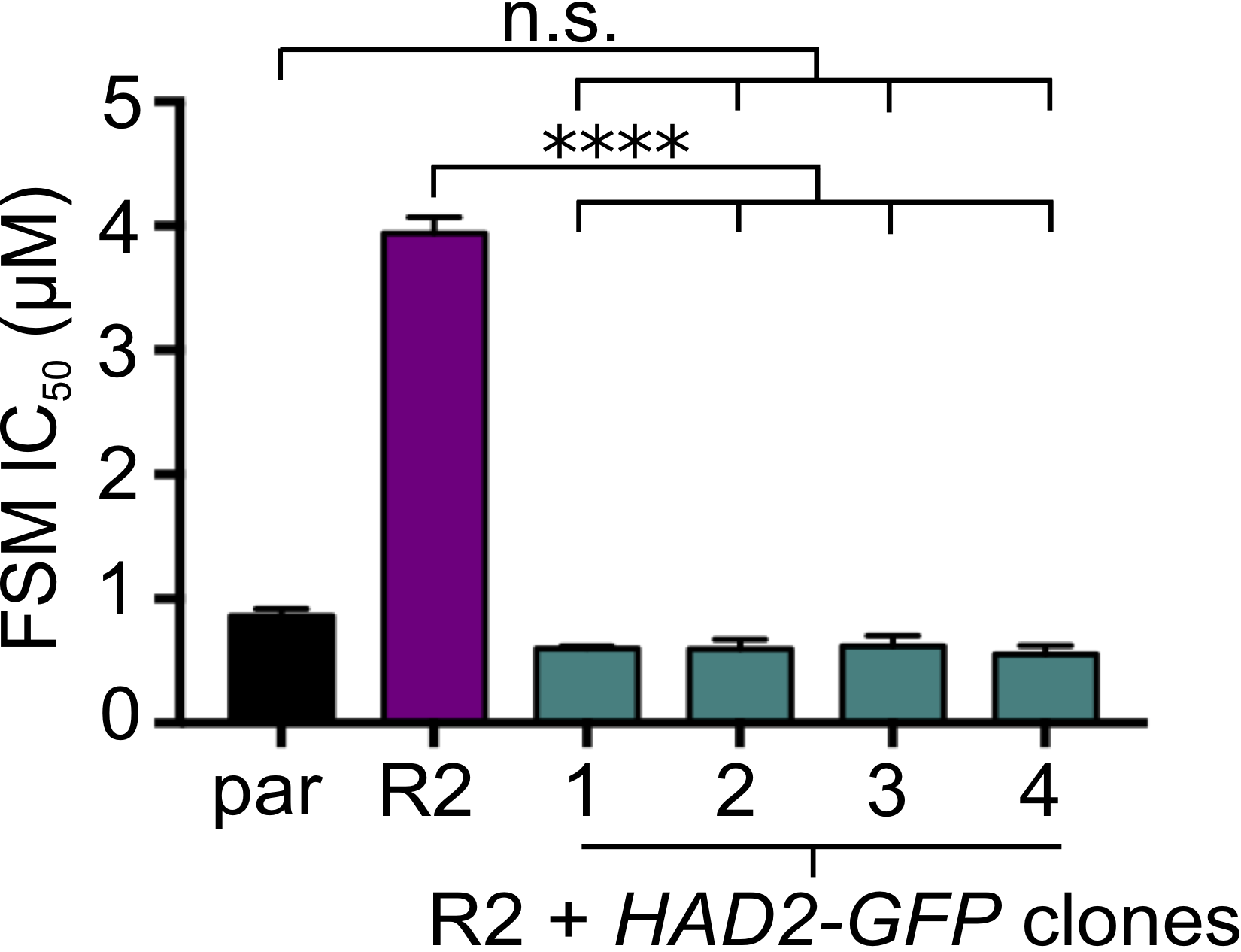

Supplement: FIG S2 [file mbo006184175sf2.tif]

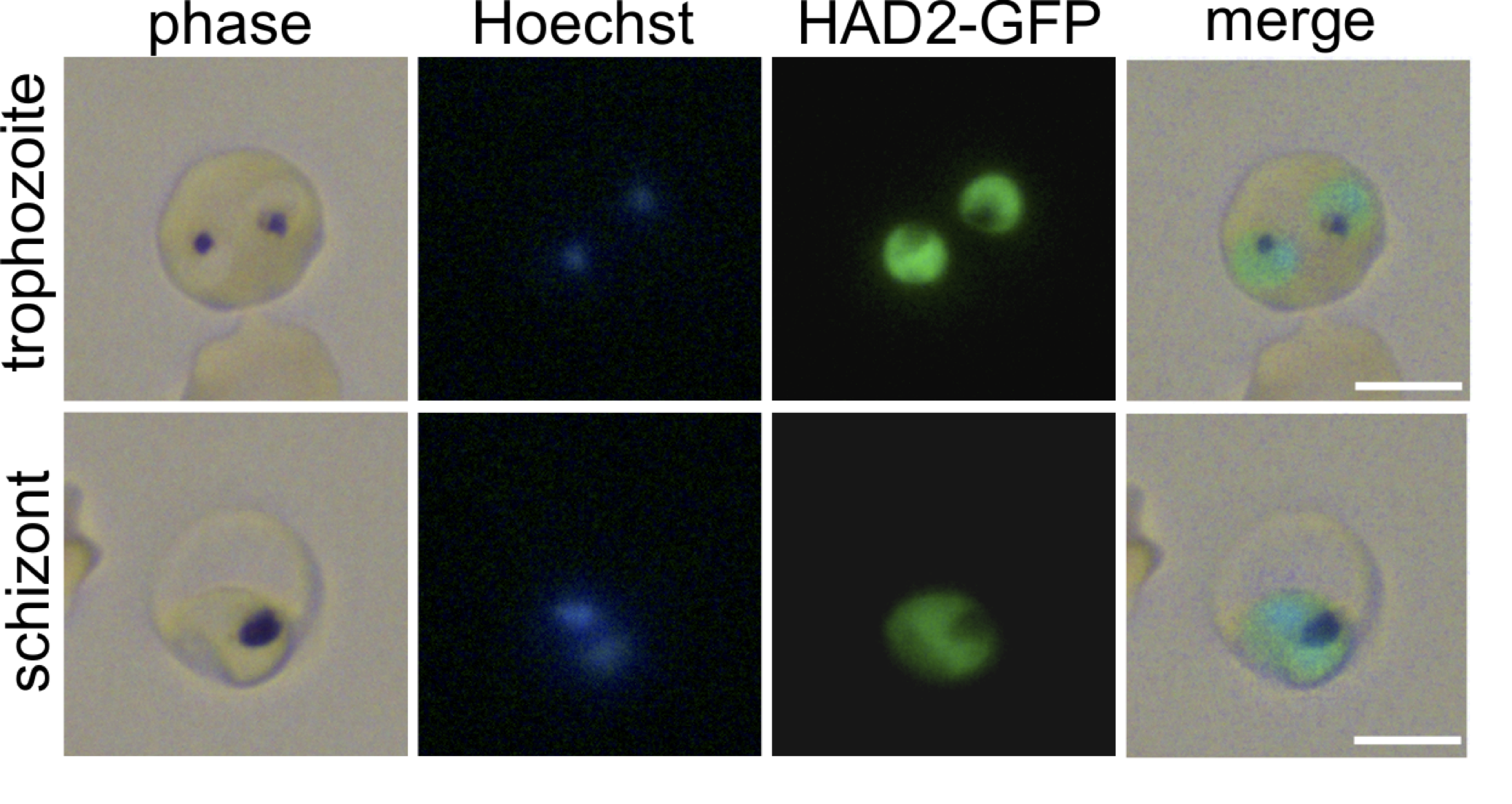

Supplement: FIG S3 [file mbo006184175sf3.tif]

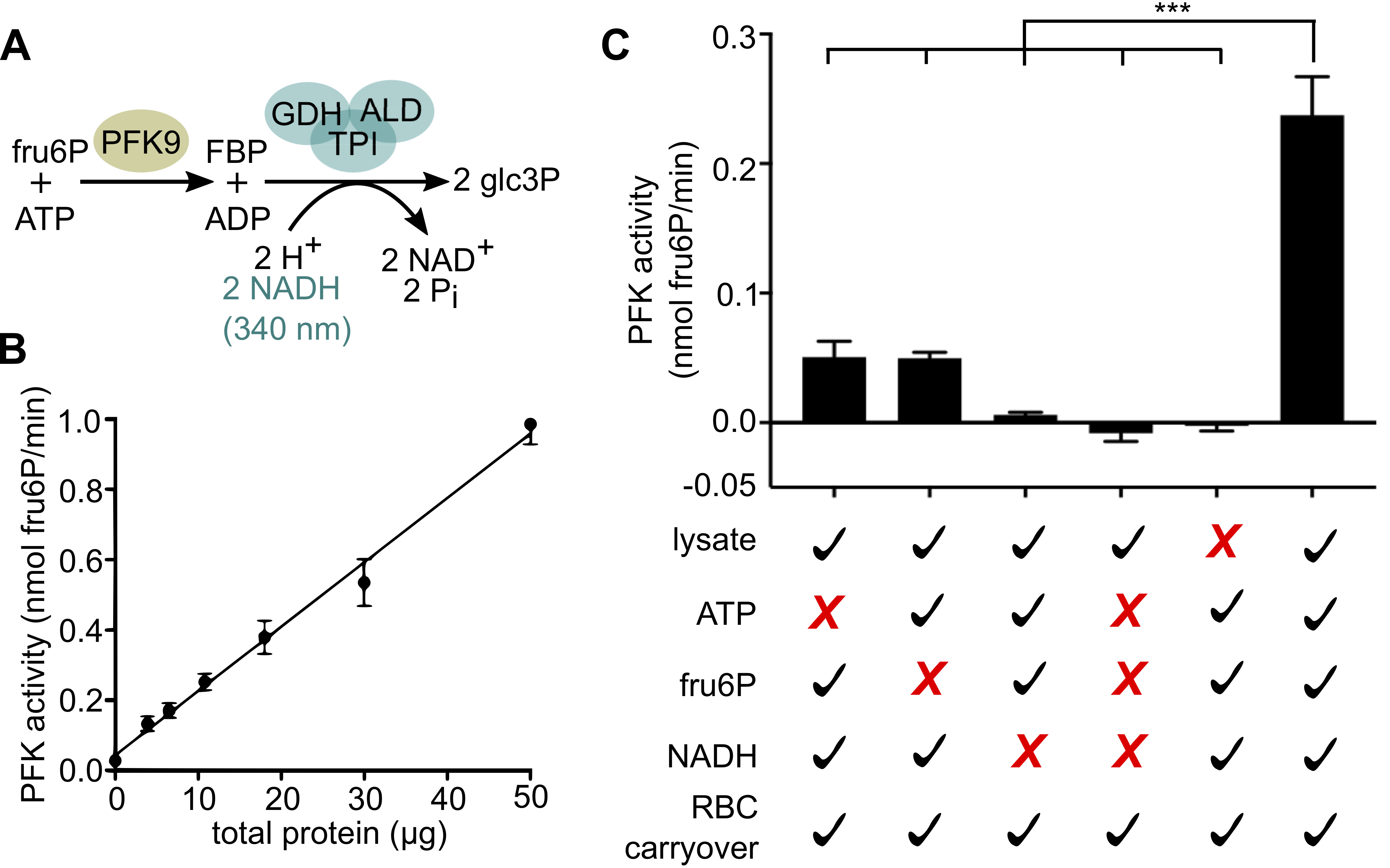

Supplement: FIG S4 [file mbo006184175sf4.tif]

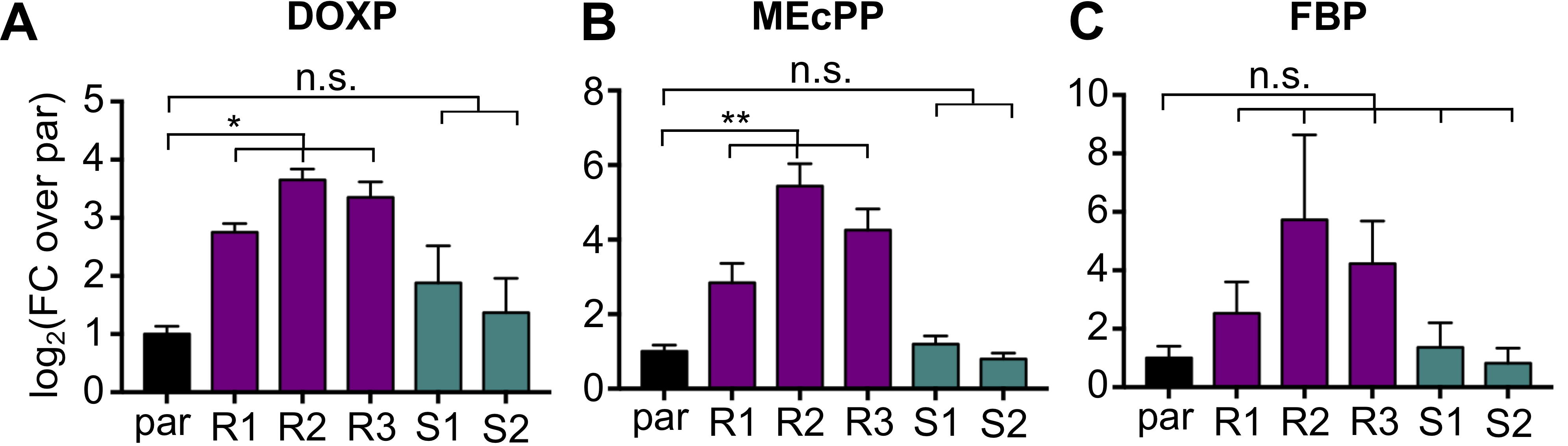

Supplement: FIG S5 [file mbo006184175sf5.tif]
