# Supplementary material for: Suppression of Drug Resistance Reveals a Genetic Mechanism of Metabolic Plasticity in Malaria Parasites
Source: mBio. 2018 Nov 13;9(6):e01193-18. doi: 10.1128/mBio.01193-18 (PMC6234871; doi:10.1128/mBio.01193-18)
Supplement: DATA SET S1 [file mbo006184175sd1.pdf]

Dataset S1. Coding SNPs identified in strains used in this study. SNPs were called and filtered as outlined in the Methods section. SNPs found in variant antigen loci such as var, rifins, stevor, etc. were removed. Columns are labeled by strain name. Variants present in the strain are indicated by read coverage at the variant site. The HAD2 SNP is highlighted in purple and the PFK9 SNP is highlighted in teal.

E2 (uncloned)	E2-R1	E2-R2	E2-R3	E2-S1	E2-S2	Chromo	Base	Ref	Variant	Locus	Annotation
74						1	162370	C	A	PFA0180w	ATP-dependent RNA helicase, putative
80						1	162391	C	A	PFA0180w	ATP-dependent RNA helicase, putative
12						1	167330	G	A	PFA0185w	L-seryl-tRNA(Sec) kinase, putative (PSTK)
22	18	8	12	19	8	1	317666	G	T	PFA0380w	serine/threonine protein kinase, putative
48						1	319169	G	T	PFA0380w	serine/threonine protein kinase, putative
16						1	408174	*	+AA/+AA	PFA0515w	phosphatidylinositol-4-phosphate 5-kinase
					58	1	409243	*	-A/-A	PFA0515w	phosphatidylinositol-4-phosphate 5-kinase
				6		1	439714	*	-GAATTG/ -GAATTG	PFA0550w	conserved Plasmodium protein, unknown function (GEXP19)
29						1	447914	C	A	PFA0570w	conserved Plasmodium protein, unknown function
			7			2	264780	*	-T/-TT	PFB0285c	conserved Plasmodium protein, unknown function
					37	2	756712	*	-A/-A	PFB0870w	conserved Plasmodium protein, unknown function
78						3	210273	A	T	PFC0195w	conserved Plasmodium protein, unknown function
72						3	210301	G	A	PFC0195w	conserved Plasmodium protein, unknown function
52						3	210337	G	A	PFC0195w	conserved Plasmodium protein, unknown function
66						3	210371	A	T	PFC0195w	conserved Plasmodium protein, unknown function
67						3	210379	G	A	PFC0195w	conserved Plasmodium protein, unknown function
17						3	211024	C	A	PFC0195w	conserved Plasmodium protein, unknown function
				8		3	224549	*	-A/-A	PFC0220w	conserved Plasmodium membrane protein, unknown function
41						3	226832	C	A	PFC0220w	conserved Plasmodium membrane protein, unknown function
				30	33	3	448056	*	-A/-A	PFC0435w	parasite-infected erythrocyte surface protein (PIESP1)
205						3	515894	*	-TTCTACACA/ -TTCTACACA	PFC0515c	TPR domain containing protein
	113					3	533827	*	-GAACGAAGACGACA/ -GAACGAAGACGACA	PFC0540w	hypothetical protein
13						3	567408	G	T	PFC0582c	vesicle transport v-SNARE protein, putative
44						3	759013	C	T	PFC0815c	conserved Plasmodium protein, unknown function
30						3	886949	T	A	PFC0940c	conserved Plasmodium protein, unknown function
30						3	886950	T	A	PFC0940c	conserved Plasmodium protein, unknown function
42						3	887274	C	T	PFC0940c	conserved Plasmodium protein, unknown function

	651					4	111496	*	-TGATGCTGT/ -TGATGCTGT	PFD0080c	Plasmodium exported protein (PHISTb), unknown function (PfD80)
	9					4	345450	T	A	PFD0320c	conserved Plasmodium protein, unknown function
		6				4	347530	T	A	PFD0320c	conserved Plasmodium protein, unknown function
		5				4	347664	A	T	PFD0320c	conserved Plasmodium protein, unknown function
		29				4	411266	*	-T/-TT	PFD0405c	zinc finger, RAN binding protein, putative
		14		8		4	760310	*	-T/-TT	PFD0835c	LETM1-like protein, putative
			25			4	934515	*	-AAT/-AAT	PFD0985w	transcription factor with AP2 domain(s) (ApiAP2)
			14			5	189990	*	-TT/-TT	PFE0235c	conserved Plasmodium protein, unknown function
				5		5	318360	*	-T/-T	PFE0380c	nuclear protein localization protein 4, putative (NPL4)
			7			5	369272	*	-AAAAA/-AA	PFE0440w	conserved Plasmodium protein, unknown function
			43	37		5	576911	*	-T/-T	PFE0675c	deoxyribodipyrimidine photo-lyase, putative
			8			5	577826	*	-T/-T	PFE0675c	deoxyribodipyrimidine photo-lyase, putative
		6				5	596880	*	+T/+T	PFE0710w	conserved Plasmodium protein, unknown function
	17					5	667603	*	-A/-A	PFE0805w	cation-transporting ATPase 1 (ATPase1)
			165			5	722130	*	+TCATCATCT/ +TCATCATCT	PFE0860c	conserved Plasmodium protein, unknown function
28						5	899148	*	-ATTATTATT/-ATT	PFE1105c	conserved Plasmodium protein, unknown function
		5				5	1093982	*	+A/+A	PFE1320w	conserved Plasmodium protein, unknown function
	20					5	1275261	*	-T/-TT	PFE1555c	conserved Plasmodium protein, unknown function
					10	6	346631	*	-A/-A	PFF0410w	conserved Plasmodium protein, unknown function
					366	6	571884	*	-GACGATAATGGT/ -GACGATAATGGT	PFF0670w	transcription factor with AP2 domain(s) (ApiAP2)
					11	6	1286488	*	-AA/-AAAA	PFF1490w	bifunctional methylenetetrahydrofolate dehydrogenase /cyclohydrolase, putative
14						7	228438	*	-T/-TT	MAL7P1.16	conserved Plasmodium membrane protein, unknown function
				17		7	417751	*	-AAA/-AAA	MAL7P1.23	RAP protein, putative
		9				7	473107	*	-T/-T	PF07_0037	Cg2 protein (CG2)
					6	8	247965	*	-A/-AA	MAL8P1.140	methionine aminopeptidase 1c, putative (METAP1c)
			9			8	319170	*	-TAT/-TAT	MAL8P1.203	serine/threonine protein kinase, FIKK family (FIKK8)
					15	8	405263	*	-T/-T	MAL8P1.125	tyrosine--tRNA ligase (TyrRS)
			10			8	522617	*	+AA/+AA	PF08_0098	ABC1 family, putative (ABCK1)
		5				8	640605	*	-T/-T	MAL8P1.97	probable protein, unknown function
31						8	702618	*	+AATAAT/+AATAAT	MAL8P1.88	conserved Plasmodium protein, unknown function
71						8	876329	G	A	PF08_0051	conserved Plasmodium protein, unknown function

11						8	891563	T	C	MAL8P1.61	conserved Plasmodium protein, unknown function
		57			42	8	1039679	*	-T/-T	PF08_0033	membrane skeletal protein IMC1-related
	14					8	1196609	*	-T/-T	PF08_0013	conserved Plasmodium membrane protein, unknown function
	28				41	8	1204618	*	-AAAAA/-AAAAA	PF08_0012	SET domain protein, putative (SET3)
		31				9	602621	*	-T/-T	PFI0690c	conserved Plasmodium protein, unknown function
				227	374	9	651216	G	A	PFI0755c	6-phosphofructokinase (PFK9)
				6		9	1096029	*	-T/-TT	PFI1315c	conserved Plasmodium protein, unknown function
	12					9	1321316	*	-AA/-AAA	PFI1615w	conserved Plasmodium protein, unknown function
				18		10	301891	*	-TTA/-TTA	PF10_0075	transcription factor with AP2 domain(s) (ApiAP2)
43		58				10	466713	*	-AAA/-A	PF10_0117	E3 ubiquitin-protein ligase, putative
27						10	644179	*	-T/-TT	PF10_0157	conserved Plasmodium protein, unknown function
		58				10	739985	*	-TTTT/-TT	PF10_0179	PHF5-like protein, putative
				20		10	1089492	*	-T/-T	PF10_0253	conserved Plasmodium protein, unknown function
				6		10	1278433	*	+A/+AAA	PF10_0310	conserved Plasmodium protein, unknown function (GEXP15)
	427					10	1425207	*	-CTGAAGAAGTAG/ -CTGAAGAAGTAG	PF10_0351	probable protein, unknown function (M566)
		28				11	349350	*	-T/-T	PF11_0092	mechanosensitive ion channel protein
					13	11	564953	*	-AA/-AA	PF11_0158	conserved Plasmodium protein, unknown function
		10				11	1427107	*	-T/-TT	PF11_0375	signal recognition particle subunit SRP72, putative (SRP72)
25						12	546028	*	+AAAAAAAAAAAA AAAAAAAAAAA/ +AAAAAAAAAAAA AAAAAAAAAAA	PFL0610w	conserved Plasmodium protein, unknown function
					9	12	637511	*	-TT/-TT	PFL0755c	conserved Plasmodium protein, unknown function
39						12	959488	*	-A/-A	PFL1130c	conserved Plasmodium protein, unknown function
90	12	27	11	16	23	12	1066255	A	T	PFL1270w	haloacid dehalogenase-like hydrolase, putative (HAD2)
				9		12	1108196	*	-AA/-A	PFL1320w	conserved Plasmodium protein, unknown function
					5	12	1172218	*	-TT/-TT	PFL1395c	conserved Plasmodium protein, unknown function
					77	12	1188112	*	+AAAAAAAA/ +AAAAAAAA	PFL1405w	conserved Plasmodium membrane protein, unknown function
20						12	1236714	*	-CC/-CC	PFL1445w	conserved Plasmodium protein, unknown function
			15			12	1359282	*	-A/-A	PFL1595w	conserved protein, unknown function
			7			12	1441873	*	+T/+T	PFL1670c	conserved Plasmodium protein, unknown function
32						12	1546147	*	-T/-T	PFL1795c	conserved Plasmodium protein, unknown function
		6				12	1649846	*	-A/-A	PFL1915w	DNA gyrase subunit B (GyrB)

				8		12	1772562	*	-T/-T	PFL1995c	conserved Plasmodium protein, unknown function
	9					12	1872538	*	-A/-A	PFL2145w	conserved Plasmodium protein, unknown function
	92	94	41	62	76	12	2023643	*	-TGTTGT/-TGTTGT	PFL2385c	conserved Plasmodium protein, unknown function
		5				13	165225	*	-TT/-TT	PF13_0018	conserved Plasmodium protein, unknown function
			6			13	353229	*	-TT/-TTT	MAL13P1.37	tripartite motif protein, putative
		21				13	391451	*	-TT/-T	MAL13P1.39	conserved Plasmodium protein, unknown function
				14	9	13	406435	*	-T/-TT	MAL13P1.42	tyrosine recombinase (INT)
				9		13	742472	*	-T/-TT	MAL13P1.93	small ribosomal subunit nuclear export protein, putative
				13		13	956200	*	-T/-T	PF13_0127	conserved Plasmodium protein, unknown function
				8		13	1130368	*	-TTT/-TTT	MAL13P1.370	conserved Plasmodium membrane protein, unknown function
	32	57				13	1131964	*	+TTTTTTTTTTTTTTTT/-T	MAL13P1.370	conserved Plasmodium membrane protein, unknown function
		315				13	1465707	*	-TGGATTTGG/ -TGGATTTGG	PF13_0201	sporozoite surface protein 2 (TRAP)
					8	13	1590959	*	-T/-TT	MAL13P1.202	conserved Plasmodium protein, unknown function
40						13	1640435	*	-TAA/-TAA	PF13_0225	conserved Plasmodium protein, unknown function
				8		13	2182112	*	-A/-A	PF13_0290	conserved Plasmodium protein, unknown function
		29			17	13	2309418	*	-AA/-AAA	MAL13P1.286	conserved Plasmodium protein, unknown function
			6			14	358846	*	-A/-A	PF14_0088	aldo-keto reductase, putative
	11					14	962974	*	-TAT/-TAT	PF14_0228	conserved Plasmodium protein, unknown function
	19					14	1161423	*	-A/-AA	PF14_0273	rRNA (adenosine-2'-O-)-methyltransferase, putative
				5		14	1668898	*	-A/-A	PF14_0388	conserved Plasmodium protein, unknown function
					57	14	1975642	*	-TTGTTGTTGTTG/ -TTGTTGTTGTTG	PF14_0461	conserved Plasmodium protein, unknown function
	14					14	2085310	*	-T/-T	PF14_0485	conserved Plasmodium protein, unknown function
		6				14	2370798	*	-ATAAA/-ATAAA	PF14_0550	large ribosomal subunit nuclear export factor, putative
