# Supplementary material for: Suppression of Drug Resistance Reveals a Genetic Mechanism of Metabolic Plasticity in Malaria Parasites
Source: mBio. 2018 Nov 13;9(6):e01193-18. doi: 10.1128/mBio.01193-18 (PMC6234871; doi:10.1128/mBio.01193-18)
Supplement: TABLE S1 [file mbo006184175st1.docx]

**Supplemental Table 1. Relative levels of all metabolites in strains described in this study.** Data shown are mean and S.E.M. of the fold-change (FC) over the parental (par) strain from ≥3 independent experiments.

|  | **R1** | **R2** | **R3** | **S1** | **S2** | **par** |
| --- | --- | --- | --- | --- | --- | --- |
| **glu6P/fru6P** | 1.57 ± 0.51 | 1.60 ± 0.58 | 1.51 ± 0.55 | 1.90 ± 0.50 | 1.14 ±  0.35 | 1.00 ± 0.18 |
| **FBP** | 2.53 ±  1.08 | 5.73 ±  2.91 | 4.23 ±  1.46 | 1.36 ±  0.84 | 0.82 ±  0.51 | 1.00 ±  0.40 |
| **DHAP/gly3P** | 0.90 ±  0.48 | 0.96 ±  0.56 | 0.85 ±  0.48 | 0.64 ±  0.14 | 0.32 ±  0.12 | 1.00 ±  0.19 |
| **2PGA** | 0.74 ±  0.41 | 0.93 ±  0.54 | 0.85 ±  0.48 | 0.64 ±  0.33 | 0.35 ±  0.19 | 1.00 ±  0.21 |
| **PEP** | 0.42 ±  0.22 | 0.62 ±  0.37 | 0.50 ±  0.24 | 0.80 ±  0.40 | 0.51 ±  0.28 | 1.00 ±  0.34 |
| **DOXP** | 2.75 ±  0.15 | 3.65 ±  0.19 | 3.35 ±  0.27 | 1.88 ±  0.64 | 1.37 ±  0.59 | 1.00 ±  0.14 |
| **MEcPP** | 2.84 ±  0.52 | 5.44 ±  0.60 | 4.25 ±  0.57 | 1.20 ±  0.22 | 0.80 ±  0.16 | 1.00 ±  0.17 |
