# Supplementary material for: Suppression of Drug Resistance Reveals a Genetic Mechanism of Metabolic Plasticity in Malaria Parasites
Source: mBio. 2018 Nov 13;9(6):e01193-18. doi: 10.1128/mBio.01193-18 (PMC6234871; doi:10.1128/mBio.01193-18)
Supplement: TABLE S2 [file mbo006184175st2.docx]

**Supplementary Table 2. Primers used in this study.** Underlines indicate overhangs used for cloning.

| **Primer name** | **Purpose** | **Sequence (5’->3’)** |
| --- | --- | --- |
| HAD2_R157X_F | Genotyping *had2^R157X^* allele, detection of HAD2-GFP construct | AGGATATACTTTATTAGATGAGAC |
| HAD2_R157X_R | Genotyping *had2^R157X^* allele | ATTATATGTTGTAAATATGGTCAATT |
| PFK9_F | Amplifying *PFK9* | CTCACCACCACCACCACCATATGGATACCAAGAGTGGAGATAAAA |
| PFK9_R | Amplifying *PFK9* | ATCCTATCTTACTCACTTAGTTCATTCTTTTTCTCTGGTTTTC |
| PFK9_seq_1 | Sequencing *PFK9* | CACAGGTAATGAATTCCCAGC |
| PFK9_seq_2 | Sequencing *PFK9* | ATCATCGGCATTCTGACATAAC |
| PFK9_seq_3 | Sequencing *PFK9* | GATCTATGCGTTTTGAACAATTAG |
| PFK9_seq_4 | Sequencing *PFK9* | CCTGTTAAGCCACTTTCAATAAC |
| PFK9_seq_5 | Sequencing *PFK9* | CCCAGTTGATTCTCTATCCATTAA |
| PFK9_seq_6 | Sequencing *PFK9* | GGTCCATTTGATGCTTCGAAAC |
| PFK9_seq_7 | Sequencing *PFK9* | CGTTGTTATACTGTAAAGACTCCA |
| PFK9_seq_8 | Sequencing *PFK9* | CTAGTCCCATTTCTTTTGAAATAAGAA |
| HAD2_LIC_F | Cloning *HAD2* into vector BG1861 | CTCACCACCACCACCACCATATGGCTTCTAGTAACGATGTACA |
| HAD2_LIC_R | Cloning *HAD2* into vector BG1861 | ATCCTATCTTACTCACTTATTTTTTTTTCAAGTCAAATACTTTTTTTAA |
| HAD2_D26A_F | Site-directed mutagenesis of HAD2 | GTTATTATTAATTGCTTTTGATGGTACAT |
| HAD2_D26A_R | Site-directed mutagenesis of HAD2 | ATGTACCATCAAAAGCAATTAATAATAAC |
| DXS_F | qPCR of *DXS* (Pf3D7_1337200) mRNA | AACGTGGATAAAGTACACATTGC |
| DXS_R | qPCR of *DXS* (Pf3D7_1337200) mRNA | TGATATACTTACGGCATTTGTTGG |
| DXR_F | qPCR of *DXR* (PF3D7_1467300) mRNA | ACATGGCCTGATAGAATAAAAACA |
| DXR_R | qPCR of *DXR* (PF3D7_1467300) mRNA | TTCATTTGACGCATTTAGTACAGTT |
| betatub_F | qPCR of *beta tubulin* (PF3D7_1008700) mRNA | ATCCCATTCCCACGTTTACATT |
| betatub_R | qPCR of *beta tubulin* (PF3D7_1008700) mRNA | TCCTTTGTGGACATTCTTCCTC |
| 18S_F | qPCR of *18S rRNA* (PF3D7_0112300) | GAACGAGGAATGCCTAGTAAGCA |
| 18S_R | qPCR of *18S rRNA* (PF3D7_0112300) | TTCATCATATCTTTCAATCGGTAGGA |
| HAD2_XhoI_F | Cloning *HAD2* into vector pTEOE110 | GATCCTCGAGATGGCTTCTAGTAACGATGTACATT |
| HAD2_AvrII_R | Cloning *HAD2* into vector pTEOE110 | GATCCCTAGGTTTTTTTTTCAAGTCAAATACTTTTTTTAATAAG |
| GFP_R | Detection of HAD2-GFP construct | CCGTATGTTGCATCACCTTC |
