# Supplementary material for: Suppression of Drug Resistance Reveals a Genetic Mechanism of Metabolic Plasticity in Malaria Parasites
Source: mBio. 2018 Nov 13;9(6):e01193-18. doi: 10.1128/mBio.01193-18 (PMC6234871; doi:10.1128/mBio.01193-18)
Supplement: TEXT S1 [file mbo006184175s1.docx]

**Supplemental Materials and Methods**

**Quantitative PCR**

Total RNA was extracted from saponin-lysed parasites using the Ambion Purelink RNA Mini kit (Thermo Fisher Scientific). An on-column DNase (Qiagen) treatment was performed after the first wash step. To synthesize cDNA, one microgram of total RNA was used in reverse transcriptase reactions using the Quantitect Reverse Transcription kit (Qiagen).

PCR reactions contained Fast SYBR Green PCR Master Mix (Applied Biosystems) and 300 nM each primer. Primers used are listed in Table S2. Thermocycling was performed on an Applied Biosystems 7500 Fast RT PCR System (95 °C for 30 s, 40 cycles of 95 °C for 3 s, 60 °C for 30 s). Controls lacking reverse transcriptase and template produced no significant signal. Melt curve analysis verified that all primer sets produce single products. Relative expression levels were calculated using the ΔΔC_t_ method. All data are means calculated from independent experiments using biological and technical replicates.

**Live fluorescent microscopy**

Erythrocytes infected with HAD2-GFP parasites were stained with 10 ng/µL Hoechst 33258 and mounted under coverslips on Polysine adhesion slides (ThermoFisher). Cells were viewed on an Olympus BH8 microscope. Minimal adjustments to contrast and brightness were applied equally to all microscopy images using Inkscape and GIMP software.
